# Clinical features and nonoperative strategy for first-time infected urachal cysts in infants

**DOI:** 10.3389/fped.2026.1870300

**Published:** 2026-07-29

**Authors:** Baifeng Chen, Bingliang Li, Wei Wang, Dong Wang, Tianhua Luo, Xuhui Zhang

**Affiliations:** 1 Department of Urology, Shanxi Medical University Affiliated Children’s Hospital, Taiyuan, China; 2 Department of Neonatal Surgery, Shanxi Medical University Affiliated Children’s Hospital, Taiyuan, China; 3 Department of Pediatrics, Shanxi Medical University, Taiyuan, China

**Keywords:** infant, infection, nonoperative management, surgical timing, urachal cyst

## Abstract

**Objectives:**

To investigate the clinical characteristics and outcomes of nonoperative management in infants aged ≤12 months with first-time infected urachal cysts, with a focus on comparing treatment responses between the ≤6-month and 6–12-month age groups.

**Methods:**

A retrospective analysis was conducted on clinical data and follow-up outcomes of infants aged ≤12 months with first-time infected urachal cysts admitted between January 2012 and January 2026. All patients initially received nonoperative management (including antibiotic therapy alone, or antibiotic therapy combined with ultrasound-guided percutaneous drainage). Clinical characteristics and cyst regression were recorded. Patients were stratified by age into the ≤6-month group and the 6–12-month group for comparison. Cyst outcomes were classified into three categories: complete resolution, marked regression (≥50% reduction with residual lesions), and treatment failure (requiring surgery).

**Results:**

A total of 49 infants were enrolled. Umbilical local manifestations were the most common presenting symptoms (79.6%), and the abscess formation rate was 75.5%. The complete resolution rate was 79.6% (39/49), the marked regression rate was 8.2% (4/49), and the eventual surgical rate was 12.2% (6/49). The complete resolution rates were 77.3% and 81.5% in the ≤6-month and 6–12-month groups, respectively; however, the younger group had a significantly higher rate of percutaneous drainage (90.9% vs. 62.9%, *P* = 0.024). All infants who required surgery had their surgical indications established within 3 months after infection control.

**Conclusions:**

In infants (≤12 months of age) with first-time infected urachal cysts, the majority achieve complete cyst resolution after nonoperative management. The observation that all surgical cases clustered within 3 months after infection control warrants attention, but it should not be used as a definitive surgical decision threshold. These conclusions are based on short-term follow-up data; long-term safety requires further confirmation through extended follow-up.

## 1 Introduction

Urachal cyst represents the most prevalent type among urachal remnant anomalies, with infection being its most common complication (1, 2). Disease progression may lead to abdominal wall abscess, diffuse peritonitis, and even sepsis (3). Currently, treatment strategies for infected urachal cysts remain heterogeneous. The conventional approach advocates complete cyst excision to prevent recurrence and long-term malignant transformation; however, primary surgery during the acute infectious phase carries a higher risk of complications due to significant local tissue edema and obscured anatomical planes (4). Consequently, a staged therapeutic strategy involving initial infection control followed by interval definitive surgery is commonly employed in clinical practice (5).

Recent studies have demonstrated that asymptomatic urachal cysts in infants possess spontaneous involution potential within 6–12 months after birth (6, 7), suggesting that nonoperative management may hold unique value in this younger population. Isolated case reports have documented gradual cyst shrinkage or even complete resolution in infants with infected urachal cysts following standardized antibiotic therapy (8, 9). Nevertheless, the prevailing clinical paradigm for infected urachal cysts remains infection control followed by elective surgery. Whether infected urachal cysts can achieve favorable resolution outcomes comparable to those of asymptomatic cysts after infection control, whether regression rates differ across various observation periods, and the appropriate window for nonoperative management and optimal surgical timing remain insufficiently investigated.

Accordingly, this study presents a retrospective analysis of clinical characteristics, nonoperative outcomes, and surgical timing in infants aged ≤12 months with first-time infected urachal cysts. The aims were to determine whether favorable resolution comparable to spontaneous involution of asymptomatic cysts could be achieved following infection control, to identify whether regression rates differ across distinct observation windows, and to provide preliminary evidence for nonoperative treatment decision-making in infants with infected urachal cysts.

## 2 Materials and methods

### 2.1 Study subjects and grouping

This single-center retrospective study enrolled infants with first-time infected urachal cysts admitted to the Department of Urology, Shanxi Children's Hospital, between January 2012 and January 2026. Inclusion criteria were: (1) age ≤12 months; (2) radiological confirmation (ultrasonography or computed tomography) of urachal cyst; (3) first presentation with cyst-related infection; and (4) complete clinical and follow-up data. Exclusion criteria were: (1) prior history of urachal cyst infection or surgical intervention; (2) concurrent complex urogenital malformations or other abdominal infections; and (3) follow-up duration <12 months.

To explore potential differences in clinical characteristics and treatment outcomes across developmental stages, patients were stratified by age at admission into the ≤6-month group and the 6–12-month group. The 6-month cutoff was chosen based on literature on the natural history of asymptomatic urachal cysts (10, 11), which suggests that this time point is a critical phase for spontaneous regression of urachal remnants. However, these data come from observational studies of asymptomatic cysts. Whether this applies to infected cysts that have undergone inflammation, edema, and fibrosis lacks direct evidence. Therefore, our age stratification is exploratory, and the validity of this cutoff in infected cysts requires further verification.This study was approved by the Institutional Review Board of Shanxi Children's Hospital (IRB-KYYN-2023-010), with waiver of informed consent granted.

### 2.2 Treatment protocol and follow-up

All patients received standardized nonoperative management. In this study, “nonoperative management” was defined as all initial interventions other than surgical excision aimed at radical cyst removal. It specifically included two tiers: (1) antibiotic therapy alone; and (2) antibiotic therapy combined with ultrasound-guided percutaneous drainage.Upon diagnosis, empirical antibiotic therapy with third-generation cephalosporins was initiated immediately, with subsequent adjustment based on culture and susceptibility results of drainage secretions or aspirated fluid. For patients with abscess formation confirmed by ultrasonography, ultrasound-guided percutaneous drainage was performed in addition to antimicrobial therapy. The indications for percutaneous drainage included: (1) ultrasonographic evidence of liquefied necrotic areas within the cyst; and (2) progressive enlargement of periumbilical soft tissue erythema/edema or development of fluctuance. These indications were determined by the attending physicians based on comprehensive assessment of clinical manifestations, dynamic ultrasound findings, and laboratory results. The specific timing and procedural approach were decided jointly by ultrasonographers and urologists. Vital signs, local umbilical findings, and inflammatory markers were closely monitored throughout the treatment course. Discharge criteria included: afebrile status for ≥48 h, marked improvement or resolution of umbilical symptoms, and normalization or sustained decline of inflammatory markers.

Following discharge, patients were scheduled for regular outpatient follow-up visits at 1, 2, 3, 6, and 12 months post-discharge. Abdominal ultrasonography was performed at each visit to assess cyst size changes and recurrence. All patients were followed for at least 12 months, and the 12-month time point after infection control was used as the unified analysis node. For patients who failed nonoperative management and underwent surgery, postoperative follow-up continued at the same intervals.

### 2.3 Outcome measures

The following variables were collected: sex, age in months, body mass index (BMI); presenting symptoms including umbilical manifestations, abdominal pain, fever, dysuria, and abdominal mass; maximum cyst diameter and abscess formation; white blood cell count (WBC), C-reactive protein (CRP), and procalcitonin (PCT); performance of percutaneous drainage, total duration of antibiotic therapy, and length of hospital stay. The outcomes of nonoperative management were classified into three categories: (1) complete resolution: complete disappearance of the cyst on imaging; (2) marked regression: *a* ≥ 50% reduction in maximum diameter or volume without complete disappearance; and (3) treatment failure: <50% cyst reduction, infection recurrence, or need for surgical intervention. Outcome adjudication was performed jointly by two senior attending physicians.

### 2.4 Statistical analysis

All data were analyzed using SPSS version 26.0 (IBM Corp., Armonk, NY, USA). Continuous variables were assessed for normality using the Shapiro–Wilk test. As all continuous data in this cohort violated the assumption of normality, they are presented as median (interquartile range, Q1–Q3) and compared between groups using the Mann–Whitney *U*-test. Categorical variables are expressed as frequency (percentage) and compared using the chi-square test or Fisher's exact test (when expected cell frequencies were <5). All tests were two-tailed, with *P* < 0.05 considered statistically significant. No correction for multiple comparisons was applied; *P* values are presented primarily as a reference for trends. The 95% confidence intervals (CI) were calculated using the Clopper-Pearson exact method.

## 3 Results

### 3.1 Overall clinical characteristics

**Figure 1:**
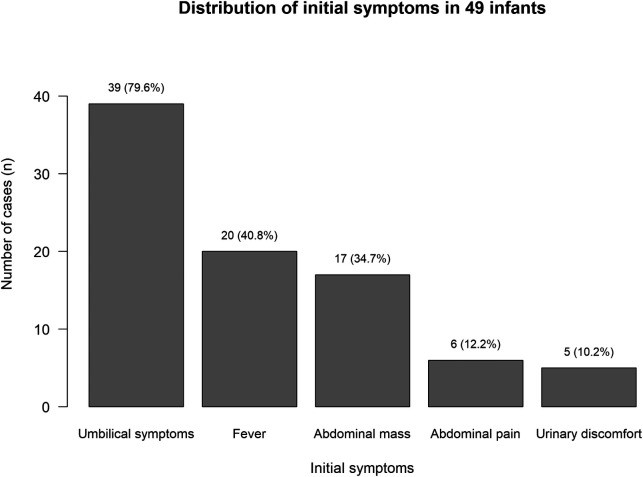
Bar chart of presenting symptom distribution in the 49 infants.

A total of 49 infants aged ≤12 months with first-time infected urachal cysts were enrolled, comprising 24 males and 25 females. The median age was 8 months, and the median body mass index was 16.74 kg/m^2^. The most common presenting symptoms were local umbilical manifestations including erythema and discharge (39 cases, 79.6%), followed by fever (20 cases, 40.8%), abdominal mass (17 cases, 34.7%), abdominal pain (6 cases, 12.2%), and dysuria (5 cases, 10.2%). The majority of infants presented with ≥2 concurrent symptoms. The median maximum cyst diameter was 3.8 cm. The median white blood cell count at admission was 16.31 × 10^9^/L, and the median C-reactive protein level was 17.47 mg/L. The abscess formation rate was 75.5% (37/49). During treatment, 37 patients (75.5%) underwent ultrasound-guided percutaneous drainage. The median total duration of antibiotic therapy and length of hospital stay were both 9 days. Complete cyst resolution was achieved in 39 cases (79.6%), marked regression (≥50% reduction with residual lesions) in 4 cases (8.2%), and 6 patients (12.2%) eventually underwent surgical excision, with complete cyst disappearance confirmed postoperatively in all cases (Table 1, Figure 1). The actual follow-up duration ranged from 1 to 14 years.

**Table 1:** Overall clinical characteristics of the 49 infants.

Variable	Result
Sex (male/female)	24/25
Age (months), M (Q1, Q3)	8 (2,10)
BMI, M(Q1,Q3)	16.74 (14.22,17.64)
Presenting symptoms, *n* (%)	
Umbilical manifestations (erythema/discharge)	39 (79.6%)
Abdominal pain	6 (12.2%)
Fever	20 (40.8%)
Dysuria	5 (10.2%)
Abdominal mass	17 (34.7%)
Maximum cyst diameter (cm), M (Q1, Q3)	3.8 (3.0,5.3)
Admission WBC (×10^9^/L), M (Q1, Q3)	16.31 (10.71,21.57)
Admission CRP (mg/L), M (Q1, Q3)	17.47 (1.02,32.39)
Admission PCT (ng/mL), M (Q1, Q3)	0.00 (0.00,0.12)
Percutaneous drainage performed, *n* (%)	37 (75.5%)
Total duration of antibiotic therapy (days), M (Q1, Q3)	9 (5,11)
Length of hospital stay (days), M (Q1, Q3)	9 (6,13)
Outcomes, *n* (%)	
Complete resolution	39 (79.6%)
Marked regression (≥50% reduction with residual lesions)	4 (8.2%)
Treatment failure (eventual surgery)	6 (12.2%)

### 3.2 Age-stratified differences

**Figure 2:**
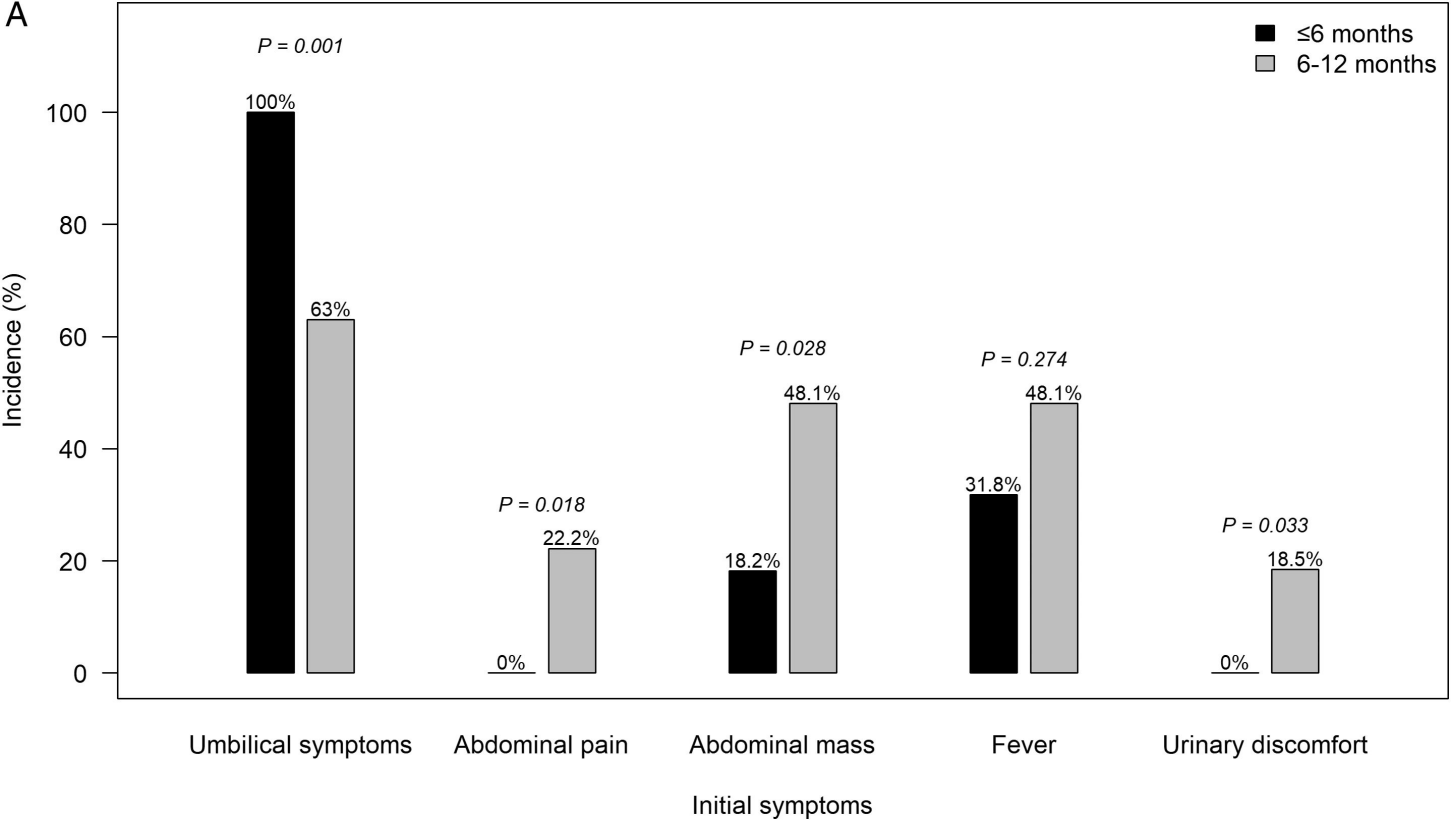

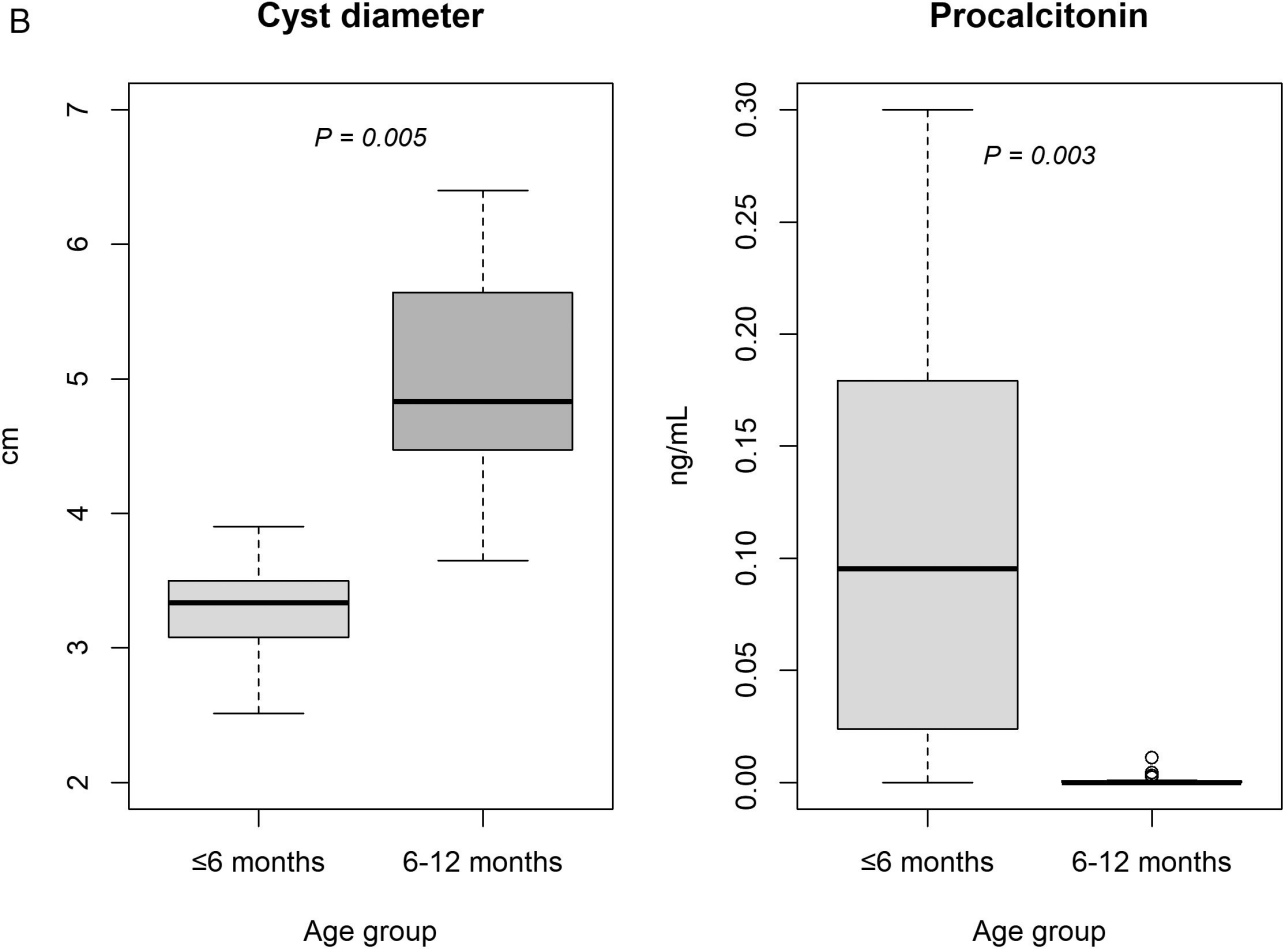
**(A)** comparison of presenting symptom incidence between age groups in infants with infected urachal cysts. **(B)** Comparison of cyst diameter and procalcitonin levels between age groups in infants with infected urachal cysts.

Patients were divided into the ≤6-month group (22 cases) and the 6–12-month group (27 cases). The incidence of umbilical manifestations was significantly higher in the ≤6-month group (*P* = 0.001), whereas abdominal pain, dysuria, and abdominal mass were more common in the 6–12-month group (all *P* < 0.05). The maximum cyst diameter was significantly smaller in the ≤6-month group (*P* = 0.005). No significant differences were observed in white blood cell count or C-reactive protein levels between the two groups (both *P* > 0.05); however, procalcitonin levels were higher in the ≤6-month group (*P* = 0.003). The rate of percutaneous drainage was higher in the ≤6-month group (90.9%) than in the 6–12-month group (62.9%, *P* = 0.024). The complete resolution rates were 77.3% (17/22) and 81.5% (22/27) in the two groups, respectively. The marked regression rates were 9.1% (2/22) and 7.4% (2/27), and the eventual surgical rates were 13.6% (3/22) and 11.1% (3/27). The differences were not statistically significant. Each group had 3 patients who eventually underwent surgery (Table 2, Figures 2A,B).

**Table 2:** Comparison of clinical characteristics and treatment outcomes between age groups in infants with infected urachal cysts.

Variable	≤6-month group (*n* = 22)	6–12-month group (*n* = 27)	Statistic Value	*P*
Sex, *n* (%)				
Male	10 (45.5%)	14 (51.9%)	0.199	0.656
Female	12 (54.5%)	13 (48.1%)		
Age (months), M (Q1, Q3)	1 (1,4)	10 (9,11)	6.034	0.001
BMI, M(Q1,Q3)	14.22 (13.68,17.54)	17.04 (16.01,17.74)	1.870	0.062
Presenting symptoms, *n* (%)				
Umbilical manifestations (erythema/discharge)	22 (100.0%)	17 (62.9%)	–	0.001
Abdominal pain	0 (0.0%)	6 (22.2%)	–	0.018
Fever	7 (31.8%)	13 (48.1%)	1.338	0.274
Dysuria	0 (0.0%)	5 (18.5%)	–	0.033
Abdominal mass	4 (18.2%)	13 (48.1%)	–	0.028
Maximum cyst diameter (cm), M (Q1, Q3)	3.3 (2.4,3.9)	5.0 (3.3,6.4)	2.803	0.005
Admission WBC (×10⁹/L)	16.13 (10.65,23.39)	17.36 (11.81,21.29)	0.422	0.673
Admission CRP (mg/L)	4.14 (0.50,23.91)	22.25 (9.21,40.76)	1.443	0.149
Admission PCT (ng/mL)	0.09 (0.00,0.15)	0.00 (0.00,0.07)	2.979	0.003
Percutaneous drainage performed, *n* (%)	20 (90.9%)	17 (62.9%)	–	0.024
Total duration of antibiotic therapy (days), M (Q1, Q3)	7 (4,10)	10 (7.5,12.5)	1.580	0.114
Length of hospital stay (days), M (Q1, Q3)	7 (4,13)	10 (8,13)	1.685	0.092
Outcomes, *n* (%)				
Complete resolution	17 (77.3%)	22 (81.5%)	0.132	0.789
Marked regression	2 (9.1%)	2 (7.4%)	–	0.675
Surgical cases	3 (13.6%)	3 (11.1%)	–	0.631

Fisher's exact test yields no test statistic; indicated by “–”.

### 3.3 Cyst outcomes and surgical timing analysis

**Figure 3:**
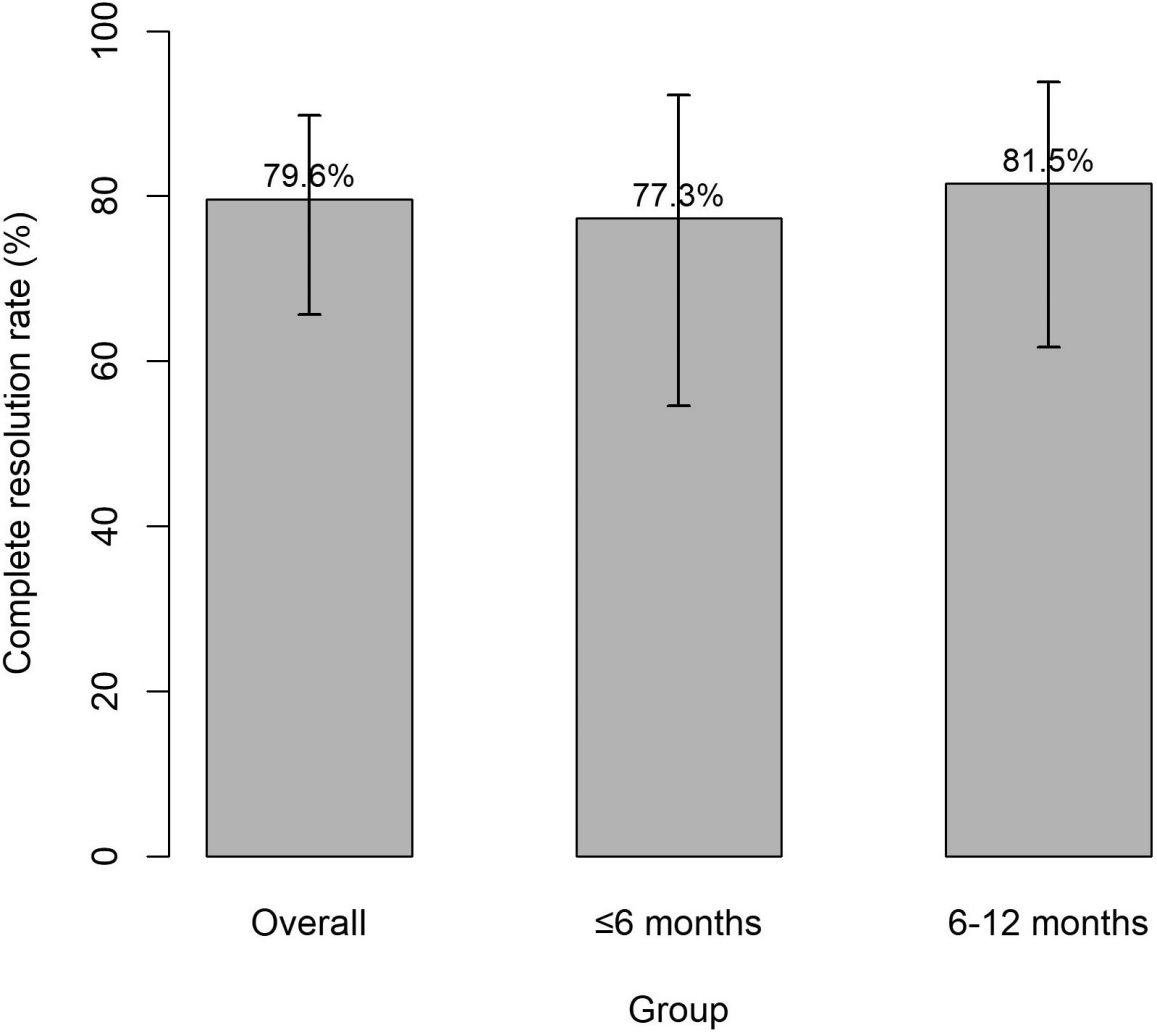
Bar chart of complete resolution rates by age group.

**Figure 4:**
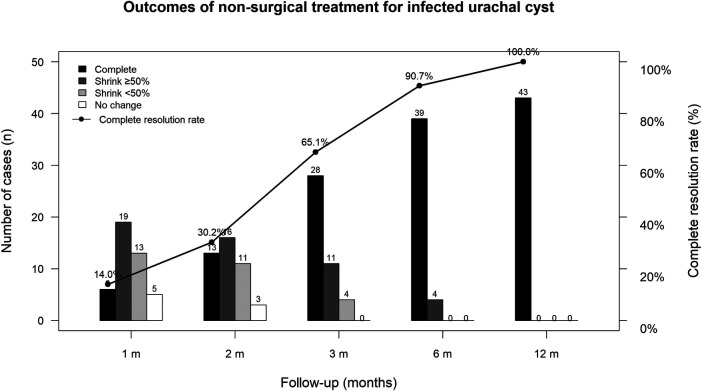
Dynamic changes in outcomes following nonoperative treatment of infected urachal cysts in infants.

In this study, all patients eventually achieved complete cyst disappearance (Among these, 39 cases achieved complete resolution after nonoperative management, and the 4 cases with marked regression also progressed to complete resolution by the 12-month follow-up; 6 cases underwent surgical excision). Follow-up analysis of the 43 patients who achieved either complete resolution or marked regression after nonoperative management showed that the rate of complete cyst disappearance gradually increased over time: 14.0% (6/43) at 1 month, 30.2% (13/43) at 2 months, 65.1% (28/43) at 3 months, 90.7% (39/43) at 6 months, and 100% (43/43) at 12 months (Table 3, Table 4, Figures 3, 4). Table 4 presents the dynamic changes in cyst size in these 43 patients following nonoperative treatment. Analysis of the entire cohort (including surgical cases) revealed that the most pronounced cyst changes occurred within the first 3 months, with stabilization thereafter. All 6 patients who required surgery underwent the procedure within 3 months after discharge, with no additional surgical cases identified beyond this timeframe.

**Table 3:** Overall outcomes of nonoperative treatment.

Group	Total cases	Complete resolution	Marked regression	Treatment failure	Complete resolution rate (%)	95% CI
≤6 months	22	17	2	3	77.3%	54.6%–92.2%
6–12 months	27	22	2	3	81.5%	61.7%–93.8%
Overall	49	39	4	6	79.6%	65.7%–89.8%

95% confidence intervals were calculated using the Clopper-Pearson exact method.

**Table 4:** Cyst changes during follow-up in infants with complete resolution or marked regression after nonoperative management (*n* = 43).

Follow-up timepoint	Complete resolution, *n* (%)	≥50% reduction, *n* (%)	<50% reduction, *n* (%)	No change/enlargement, *n* (%)	Total
1 month	6 (14.0%)	19 (44.2%)	13 (30.2%)	5 (11.6%)	43
2 months	13 (30.2%)	16 (37.2%)	11 (25.6%)	3 (7.0%)	43
3 months	28 (65.1%)	11 (25.6%)	4 (9.3%)	0 (0.0%)	43
6 months	39 (90.7%)	4 (9.3%)	0 (0.0%)	0 (0.0%)	43
12 months	43 (100.0%)	0 (0.0%)	0 (0.0%)	0 (0.0%)	43

### 3.4 Characteristics of treatment failures

A total of 6 patients experienced nonoperative treatment failure (3 in the ≤6-month group and 3 in the 6–12-month group), with ages ranging from 1 to 12 months and maximum cyst diameters from 2.7 to 7.3 cm. Four patients (66.7%) had previously undergone percutaneous drainage. The predominant failure pattern was persistent cyst (4 cases, 66.7%), followed by infection recurrence (2 cases, 33.3%). The time to failure (defined as the interval from infection control to the decision for surgery) in all cases occurred within 3 months, ranging from 1 to 3 months, with no surgical interventions required beyond this period (Table 5).

**Table 5:** Clinical characteristics of patients with nonoperative treatment failure (*n* = 6).

Variable	Patient 1	Patient 2	Patient 3	Patient 4	Patient 5	Patient 6
Age (months)	1	4	12	5	9	11
Maximum cyst diameter (cm)	2.7	4.7	4.8	5.6	3.6	7.3
Percutaneous drainage performed	No	Yes	Yes	Yes	No	Yes
Persistent cyst	Yes	Yes	Yes	No	Yes	No
Infection recurrence	No	No	No	Yes	No	Yes
Time to failure (months)	1	3	3	2	2	2

## 4 Discussion

The optimal treatment strategy for infected urachal cysts remains controversial. The conventional approach advocates complete cyst excision to prevent recurrence and long-term malignant transformation. However, during the acute infectious phase, significant local tissue edema and obscured anatomical planes increase the risk of complications from primary surgery (4). Therefore, a staged strategy—initial antibiotic therapy followed by delayed radical resection after inflammation subsides—is commonly used in clinical practice (12, 13). Our study showed that in infants aged ≤12 months with first-time infected urachal cysts, the complete resolution rate after nonoperative management was 79.6% (39/49), and only 12.2% (6/49) eventually required surgical intervention. This finding suggests that prioritizing observation and follow-up after infection control, with surgery reserved for patients with persistent cysts or recurrent infection, is a feasible strategy in this population. Our patients received intravenous antibiotics followed by sequential oral therapy after discharge, with a median total duration of 9 days, which is consistent with the step-down principle of pediatric antibiotic stewardship. However, due to the retrospective design, we could not precisely separate the days of intravenous vs. oral administration, which is one of the methodological limitations.

Cyst resolution in our cohort was achieved on the basis of antibiotic and drainage interventions, rather than true spontaneous involution. The pathophysiological process can be summarized as “infection control, cavity collapse, and fibrotic obliteration”: antibiotic therapy controls local and systemic inflammation (14), while percutaneous drainage evacuates pus, reduces intracystic pressure, and promotes apposition of the cyst walls, creating conditions for obliteration of the remnant urachal structure. Previous studies have confirmed that asymptomatic urachal cysts have a certain potential for spontaneous involution during the first year of life, especially within the first 6 months, with reported natural resolution rates up to 80% (15). Our study observed similar resolution rates in infected cysts, suggesting that even after infection, inflammation, and abscess formation, most cysts can achieve favorable outcomes after active nonoperative management. This is consistent with reports by other people (16), supporting a trial of nonoperative management as the initial approach for first-time infected urachal cysts in infancy.

All 6 patients who eventually underwent surgery in our cohort had their surgical indications established within 3 months after infection control due to cyst recurrence or persistence, and no additional surgical cases were identified beyond this time point. This observation suggests that 3 months may be a time point worthy of further exploration. However, this finding is based on only 6 treatment failure cases, with limited sample size and insufficient statistical power, far from sufficient to establish a definitive clinical decision threshold. This observation should be regarded as a preliminary trend for generating clinical hypotheses rather than a definitive rule for surgical timing. Until larger-scale, prospective validation is available, we suggest that clinicians consider this observation alongside dynamic cyst changes, risk of infection recurrence, and individual patient factors when assessing the need for surgery.

Age-stratified analysis showed that the complete resolution rates were 77.3% and 81.5% in the ≤6-month and 6–12-month groups, respectively, and the eventual surgical rates were 13.6% and 11.1%, with no statistically significant differences. However, the ≤6-month group had a significantly higher rate of percutaneous drainage than the 6–12-month group, indicating that the similar resolution outcomes were achieved under different treatment intensities, rather than under a uniform intervention protocol. A likely explanation for this difference is that younger infants have thinner abdominal walls and weaker umbilical remnants with compromised local barrier function, making infection more prone to deep extension and increasing the risk of disease progression. Consequently, clinicians tend to perform early percutaneous drainage to control local infection promptly. This essentially reflects a clinical trade-off in decision-making that integrates age and disease severity, rather than a result of randomized allocation. Therefore, the similar cyst resolution rates between the two groups should not be simplistically interpreted as “age does not affect treatment response.” A more reasonable interpretation is that under more aggressive drainage intervention in the younger group, resolution outcomes were comparable to those in the older group. Whether age is an independent factor influencing treatment response cannot be definitively determined in this study due to inherent treatment bias in the retrospective design, and requires validation in prospective studies with standardized treatment protocols.

The 6-month cutoff used in this study was based mainly on literature data on spontaneous involution of asymptomatic urachal cysts (7, 15, 17), which reported higher spontaneous resolution rates within the first 6 months. However, the cysts in our study were infected and histologically different from asymptomatic cysts. Infection can cause local tissue edema, inflammatory cell infiltration, and fibrosis, which may substantially alter the involution and repair process. Therefore, whether the developmental regression pattern of asymptomatic cysts can be directly extrapolated to infected cysts lacks direct biological evidence. The 6-month stratification in this study should be regarded as an exploratory grouping strategy based on existing literature, and the interpretative value of between-group differences remains primarily descriptive and exploratory. Current literature also shows divergence regarding the age cutoff for nonoperative management of infected urachal cysts in infancy: some studies use 6 months as the cutoff for observation, while others support extending observation up to 12 months (7, 9). Our data suggest that, under the condition of first-time infection, most infants up to 12 months of age can achieve favorable outcomes after nonoperative management, but the independent prognostic value of age itself remains unclear.

This study has several limitations. First, it is a single-center retrospective design with inherent selection and information biases. Drainage decisions and follow-up protocols were influenced by institutional experience, and no quantitative scoring tool was available to assist decision-making. In addition, the age-stratified analysis was confounded by treatment bias: the ≤6-month group had a significantly higher drainage rate than the 6–12-month group, and the similar resolution outcomes between groups cannot be directly attributed to age. Furthermore, the 6-month cutoff was extrapolated from literature on asymptomatic cysts, and its biological validity in infected cysts has not been established, so the age stratification is only exploratory. Moreover, the failure group comprised only 6 cases, and the observation that all failures clustered within 3 months is based on a limited sample with insufficient stability. Second, although the follow-up duration ranged from 1 to 14 years, it remains insufficient to fully assess long-term recurrence and malignant transformation risks. We also could not precisely separate the days of intravenous vs. oral antibiotic administration. This study did not include a pure observation control group, so we could not strictly distinguish whether cyst resolution was entirely due to therapeutic intervention. However, non-intervention during active infection would be ethically unacceptable. Therefore, “post-treatment cyst resolution” is a reasonable description of real-world clinical practice. These limitations warrant verification through multicenter, prospective studies.

## 5 Conclusions

In infants (≤12 months of age) with first-time infected urachal cysts, the majority achieve complete cyst resolution after nonoperative management with antibiotic therapy combined with drainage, suggesting that nonoperative management is safe and effective. However, “marked regression (≥50% reduction with residual lesions)” is not equivalent to cure, as the residual cystic wall carries a long-term risk of malignant transformation. The observation that all surgical cases were clustered within 3 months after infection control warrants attention, but due to the limited number of cases, it should not be used as a definitive decision threshold. These conclusions are based on short-term follow-up data; long-term safety requires further confirmation through extended follow-up.

## Data Availability

The original contributions presented in the study are included in the article/Supplementary Material, further inquiries can be directed to the corresponding author/s.

## References

[1] Ashley RA Inman BA Routh JC Rohlinger AL Husmann DA Kramer SA. Urachal anomalies: a longitudinal study of urachal remnants in children and adults. J Urol. (2007) 178(4 Pt 2):1615–8. 10.1016/j.juro.2007.03.194 17707039

[2] Parada Villavicencio C Adam SZ Nikolaidis P Yaghmai V Miller FH. Imaging of the urachus: anomalies, complications, and mimics. Radiographics. (2016) 36(7):2049–63. 10.1148/rg.2016160062 27831842

[3] Schurmans RCP Olij B Kool M Weinans CRGJ Visschers RGJ Zijta FM. Infected urachal cyst in an adult patient. J Clin Ultrasound. (2024) 52(9):1490–4. 10.1002/jcu.23791 39198024

[4] Gelikman DG Ibanez KR Ghattas YS Craver EC Casas-Melley AT Ellsworth P. Management of urachal anomalies in pediatric patients: a comparison of treatment strategies between pediatric urology and general surgery. J Pediatr Urol. (2024) 20(1):75.e1–8. 10.1016/j.jpurol.2023.09.013 37802719

[5] Stopak JK Azarow KS Abdessalam SF Raynor SC Perry DA Cusick RA. Trends in surgical management of urachal anomalies. J Pediatr Surg. (2015) 50(8):1334–7. 10.1016/j.jpedsurg.2015.04.020 26227313

[6] Tanaka A Fujii T Katami H Ishikawa R Haba R Shimono R. Omphalomesenteric ducts and urachal remnants: a retrospective study and case series. Cureus. (2024) 16(7):e63877. 10.7759/cureus.63877 39099973 PMC11298018

[7] Ueno T Hashimoto H Yokoyama H Ito M Kouda K Kanamaru H. Urachal anomalies: ultrasonography and management. J Pediatr Surg. (2003) 38(8):1203–7. 10.1016/s0022-3468(03)00268-9 12891493

[8] Zieger B Sokol B Rohrschneider WK Darge K Tröger J. Sonomorphology and involution of the normal urachus in asymptomatic newborns. Pediatr Radiol. (1998) 28(3):156–61. 10.1007/s002470050318 9561533

[9] Lipskar AM Glick RD Rosen NG Layliev J Hong AR Dolgin SE. Nonoperative management of symptomatic urachal anomalies. J Pediatr Surg. (2010) 45(5):1016–9. 10.1016/j.jpedsurg.2010.02.031 20438945

[10] Ghattas YS Gelikman DG Ibanez KR Ellsworth P Seth A. Current management strategies of urachal anomalies in pediatric patients: a scoping review. Frontiers in Urology. (2023) 3:1159439. 10.3389/fruro.2023.1159439 40778042 PMC12327296

[11] Zenitani M Nose S Oue T. Prevalence of urachal remnants in children according to age and their anatomic variants. Pediatr Surg Int. (2022) 38(10):1495–500. 10.1007/s00383-022-05183-2 35879470

[12] Kwok CM. Infected urachal cyst in an adult: a laparoscopic approach. Case Rep Gastroenterol. (2016) 10(2):269–74. 10.1159/000446642 27462196 PMC4939676

[13] Masuko T Nakayama H Aoki N Kusafuka T Takayama T. Staged approach to the urachal cyst with infected omphalitis. Int Surg. (2006) 91(1):52–6. 16706104

[14] Choi YJ Kim JM Ahn SY Oh JT Han SW Lee JS. Urachal anomalies in children: a single center experience. Yonsei Med J. (2006) 47(6):782–6. 10.3349/ymj.2006.47.6.782 17191305 PMC2687816

[15] Nissen M Rogge P Sander V Alrefai M Romanova A Tröbs RB. Pediatric urachal anomalies: monocentric experience and Mini-review of literature. Children. (2022) 9(1):72. 10.3390/children9010072 35053696 PMC8774176

[16] Dethlefs CR Abdessalam SF Raynor SC Perry DA Allbery SM Lyden ER. Conservative management of urachal anomalies. J Pediatr Surg. (2019) 54(5):1054–8. 10.1016/j.jpedsurg.2019.01.039 30867097

[17] Galati V Donovan B Ramji F Campbell J Kropp BP Frimberger D. Management of urachal remnants in early childhood. J Urol. (2008) 180(4 Suppl):1824–6. 10.1016/j.juro.2008.03.105 18721938

